# Novel *PRKAR1A* mutation in Carney complex: a case report and literature review

**DOI:** 10.3389/fendo.2024.1384956

**Published:** 2024-07-10

**Authors:** Huaqiang Zheng, Hong Kang, Yizhen Qiu, Liangxiao Xie, Jinzhi Wu, Pengbin Lai, Jiapeng Kang

**Affiliations:** ^1^ Department of Endocrinology, Zhangzhou Municipal Hospital, Zhangzhou Municipal Hospital Affiliated of Fujian Medical University, Zhangzhou, China; ^2^ Department of Dermatology, Zhangzhou Municipal Hospital, Zhangzhou Municipal Hospital Affiliated of Fujian Medical University, Zhangzhou, China; ^3^ Department of Neurology Critical Care Medicine, Zhangzhou Municipal Hospital, Zhangzhou Municipal Hospital Affiliated of Fujian Medical University, Zhangzhou, China; ^4^ Department of Medical Oncology, Zhangzhou Municipal Hospital, Zhangzhou Municipal Hospital Affiliated of Fujian Medical University, Zhangzhou, China

**Keywords:** Carney complex, *PRKAR1A* gene, gene mutation, case report, PPNAD

## Abstract

**Objective:**

Carney complex is a rare autosomal dominant syndrome that has been shown to be associated with inactivation due to *PRKAR1A* mutations. We revealed a novel *PRKAR1A* gene mutation in Chinese patient with Carney complex and review the literature to enhance understanding of Carney complex.

**Case presentation:**

A 23-year-old Chinese male patient with a family history cardiac myxoma was admitted to our Department of Endocrinology because of central obesity and hyperpigmentation. Physical examination revealed a maximum blood pressure of 150/93mmHg, a waist circumference of 102cm, a weight of 70kg, a height of 170cm, and a BMI of 24.22kg/m2. Additionally, there was spotty skin pigmentation on the lip mucosa, purple striae on the abdomen, thin skin on both legs, and visible veins. Blood examination revealed hypercortisolemia, decreased adrenocorticotropic hormone (ACTH) levels and failure to suppress cortisol with low and high-dose dexamethasone suppression tests. Magnetic resonance imaging (MRI) scan revealed multiple small adrenal nodules and Retroperitoneal neurogenic tumor. Genetic testing showed a novel heterozygous mutation in exon 5 of *PRKAR1A* (c.500_502 + 8delAAGGTAAGGGC). The patient underwent resection of the right adrenal gland and retroperitoneal neoplasms in 2020. Postoperative pathology following the right adrenal gland resection showed nodular hyperplasia of the adrenal cortex. The pathology from the retroperitoneal tumor resection revealed spindle cell tumors rich in pigment and cells. The patient was diagnosed as Carney complex according to Stratakis CA in 2001 guidelines. After long-term follow-up, the patient’s condition was stable, with weight loss, waist circumference reduction, significantly lower cortisol levels, and normal blood lipids.

**Conclusion:**

This case reported a Carney complex in a Chinese patient, characterized clinically by non-ACTH-dependent Cushing’s syndrome, familial recurrent cardiac myxomas, psammomatous melanotic schwannoma (PMS) and skin and mucosal pigmentation. A novel subtype of *PRKAR1A* mutation was discovered, which may affect the characteristics of the *PRKAR1A* protein and contribute to the development of Carney complex.

## 1 Introduction

**Figure 1:**
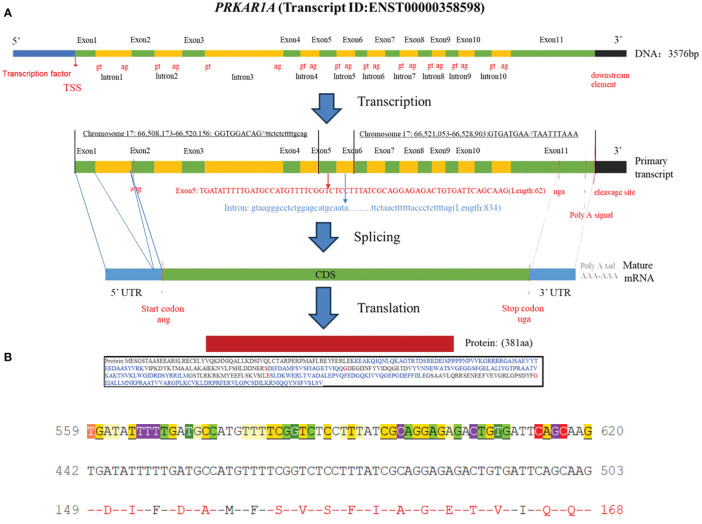
**(A)** Schematic diagram of the *PRKAR1A* gene and its encoded protein structure; **(B)** Nucleotide sequence of exon 5 of *PRKAR1A* and its translated amino acid sequence.

Carney complex is a rare autosomal dominant genetic disorder characterized by myxoma, skin pigmentation, and endocrine hyperfunction, which was first described by J. Aiden Carney in 1985 and later named Carney complex (1, 2). The epidemiology of Carney complex is still unclear, and up to now, more than 1,000 patients have been reported to have Carney complex worldwide. The pathogenesis of Carney complex has been confirmed to be related to gene mutations. Currently, the pathogenic genes reported include *PRKAR1A (* 3), *PRKACA (* 4), *PRKACB (* 5), *PDE11A (* 6), *PDE8B (* 7), etc. In addition, researchers found that the CNC2 locus on chromosome 2p16 was also associated with the pathogenesis of Carney complex (8). Among which *PRKAR1A* gene mutation was founded in more than 70% of Carney complex cases (3). *PRKAR1A* is a tumor suppressor gene located in the 17q23-24 region of the human chromosome (**Figure 1**), encoding the regulatory subunit of cAMP-dependent protein kinase A (PKA). PKA is an important signaling protein kinase involved in regulating cellular metabolism, differentiation, and proliferation (9, 10). Furthermore, PKA also is instrumental in maintaining cardiac function and is implicated in the pathogenesis of cardiac diseases (11). Mutations in the *PRKAR1A* gene lead to abnormalities in the cAMP/PKA signaling pathway, which can disrupt normal cellular function. These functional abnormalities may contribute to the development of skin pigmentation disorders and the formation of endocrine tumors, including primary pigmented nodular adrenocortical disease (PPNAD), growth hormone-secreting pituitary adenomas, thyroid tumors, and thyroid cancer in Carney complex patients. Non-endocrine tumors associated with *PRKAR1A* mutations include cardiac myxomas, cutaneous myxomas, and melanotic schwannomas.

The diagnosis of Carney complex currently relies on clinical manifestations, family history, and molecular genetic testing. Treatment options for Carney complex are tailored to individual patients based on the specific disease manifestations and may include surgical resection of tumors, radiation therapy, and pharmacological interventions. In this article, we reported a case of Carney complex presenting with non-ACTH-dependent Cushing syndrome, familial recurrent cardiac myxomas, and mucocutaneous pigmentation. Additionally, we identified a novel *PRKAR1A* mutation in this patient, which may result in defects in protein kinase function and contribute to disease pathogenesis. This finding helps enhance our understanding of the diagnosis and treatment of Carney complex and may contribute to improved clinical outcomes for affected patients.

## 2 Case report

**Figure 2:**
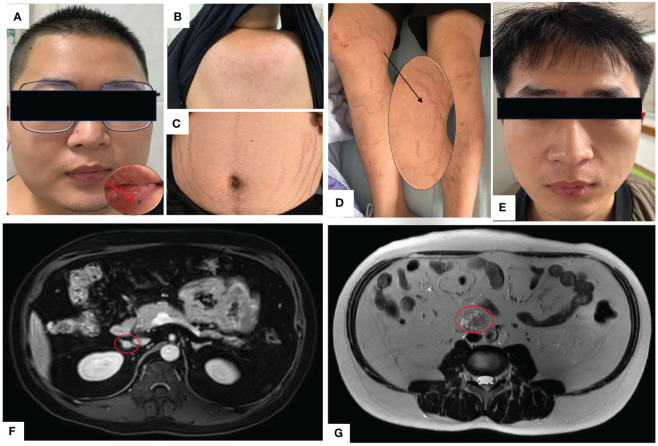
**(A)** Full moon face, pigmented moles on the lips; **(B)** Buffalo hump; **(C)** Purple lines on the abdomen; **(D)** Thin skin on both lower limbs, exposed veins; **(E)** Two years after undergoing unilateral adrenalectomy, the patient’s full moon face disappeared during follow-up. **(F)** Abdominal MRI with contrast shows multiple small nodules in bilateral adrenal glands, with the largest nodule measuring approximately 10mm×9mm. **(G)** Abdominal MRI T2-weighted imaging demonstrates a mass in the retroperitoneal space at the level of the L3-4 vertebral bodies, measuring approximately 31mm×26mm×36mm.

A 23-year-old Chinese male was admitted to our Endocrinology Department of Zhangzhou Municipal Hospital due to obesity and hyperpigmentation. Physical examination revealed that a maximum blood pressure 150/93mmHg, waist circumference 102cm, weight 70kg, height 170cm, BMI 24.22kg/m2, full moon face, several spotty skin pigmentation on the lip mucosa (**Figure 2A**), Buffalo hump (**Figure 2B**), central obesity and purple striae of the abdomen (**Figure 2C**), thin skin on both legs and bare veins (**Figure 2D**). The patient’s signs and symptoms suggest Cushing’s syndrome and should be further evaluated. He had medical history of recurrent cardiac myxoma and underwent cardiac myxoma surgery in 2006 and 2012 respectively, and recovered well after surgery. His father has a history of cardiac myxoma and underwent cardiac myxoma surgery in July 2018. The lab report indicates elevated serum cortisol levels, decreased serum adrenocorticotropic hormone (ACTH) levels, and elevated urinary free cortisol levels. The classic low-dose dexamethasone suppression test (1mg) shows no suppression of cortisol. Similarly, the high-dose dexamethasone suppression test (8mg) also indicates no suppression. Serum sex hormones and thyroid function tests are within normal limits (**Table 1** “Before diagnosis”). These findings suggest an abnormal elevation in cortisol rhythm with reduced ACTH levels, and both the low-dose and high-dose dexamethasone suppression tests indicate that cortisol is not suppressed, suggesting a non-ACTH-dependent Cushing’s syndrome.

**Table 1:** Parameters for blood tests in the long-term follow-up of patients.

Detection content	Before diagnosis	One years after unilateral adrenalectomy	Two years after unilateral adrenalectomy
Serum cortisol (0:00am)	21 (<10ug/dl)	–	–
Serum cortisol (8:00am)	23.5 (8.7-22.4ug/dl)	10.7 (8.7-22.4 ug/dl)	12.8 (8.7-22.4 ug/dl)
Serum cortisol (4:00pm)	21.1 (<10ug/dl)	–	–
Adrenocorticotropic hormone (ACTH)	0.58 (1.6-13.9pmol/L)	–	–
24 h Urinary cortisol	1431 (39-348ug/day)	460.2 (39-348ug/day)	–
Classic low-dose dexamethasone test (1mg)	Cortisol:246.4ug/L; ACTH:2.39pg/ml. Result: Not inhibited	–	–
High-dose dexamethasone suppression test (8mg)	Cortisol:296.7ug/L; ACTH:2.25pg/ml. Result: Not inhibited	–	–
Triglycerides (TG)	2.12 (0.56-1.7 mmol/L)	0.50 (0.56-1.7 mmol/L)	0.95 (0.56-1.7 mmol/L)
Total cholesterol (CHOL)	7.62 (3.0-6.0 mmol/L)	5.28 (3.0-6.0 mmol/L)	–
High Density Lipoprotein (HDL-C)	1.92 (0.8-1.7 mmol/L)	2.01 (0.8-1.7 mmol/L)	–
Low density lipoprotein (LDL-C)	4.43 (2-3.6 mmol/L)	2.91 (2-3.6 mmol/L)	2.69 (2-3.6 mmol/L)
Blood sugar	4.13 (3.5-6.0 mmol/L)	4.14 (3.5-6.0 mmol/L)	–
Free triiodothyronine (FT3)	3.98 (3.1-6.8 pmol/L)	5.38 (3.1-6.8 pmol/L)	–
Free thyroxine (FT4)	8.3 (7.5-21.1 pmol/L)	11.2 (7.5-21.1 pmol/L)	–
Thyroid-stimulating hormone 3 (TSH3)	1.22 (0.34-5.6 Uiu/mL)	2.06 (0.34-5.6 Uiu/mL)	–
Testosterone	2.05 (0.1-0.8ng/mL)	–	–
Progesterone	3.01 (1.27-2.67nmol/L)	–	–
Prolactin	8.10 (3.34-26.72ng/mL)	–	–
Growth hormone	0.157 (0.003-0.97ng/mL)	–	–

–, means that the examination was not performed in this follow-up.

The magnetic resonance imaging (MRI) scan revealed multiple small nodules in the bilateral adrenal glands, with the larger one measuring about 0.8cmX0.6cm (**Figure 2F**). The lumbar L3-4 vertebral body was occupied retroperitoneally, suggesting neurogenic tumor (**Figure 2G**). Echocardiography (post-cardiac myxoma surgery) indicates an ejection fraction of 58%, with mild mitral and tricuspid regurgitation. In October 2020, the patient underwent laparoscopic-assisted right adrenalectomy and retroperitoneal neoplasms resection in the 900th Hospital. After right adrenalectomy, postoperative pathology showed irregular tissue, medium texture, gray-brown section, golden yellow foci, and nodular hyperplasia of the adrenal cortex. Combined with blood test results, the patient was diagnosed with primary pigmented nodular adrenal diseases (PPNAD). After retroperitoneal neoplasms resection, postoperative pathology showed a nodule measuring about 3.7cm×3.1cm×2.3cm, gray-black section, soft texture, clear boundary, spindle cell tumor rich in pigment and fat. Immunohistochemical staining showed: HMB45 +++, S-100 +, Ki-67 <1%, Melan A ++, SOX10 +++, P16 +, H3K27ME3 +, PAS + (positive for lamellar calcification bodies), diagnosed as psammomatous melanotic schwannoma (PMS). Plain and enhanced MRI scans of the brain showed bilateral paraventricular infarction, with no abnormalities in the pituitary gland. Color Doppler ultrasound showed abnormal echo nodules in the left testis and calcified plaques in the right testis. Cardiac color Doppler ultrasound showed a small amount of regurgitation in the mitral and tricuspid valves, with no abnormalities in the overall systolic and diastolic function of the left ventricle. Thyroid ultrasound showed no obvious abnormalities.

**Figure 3:**
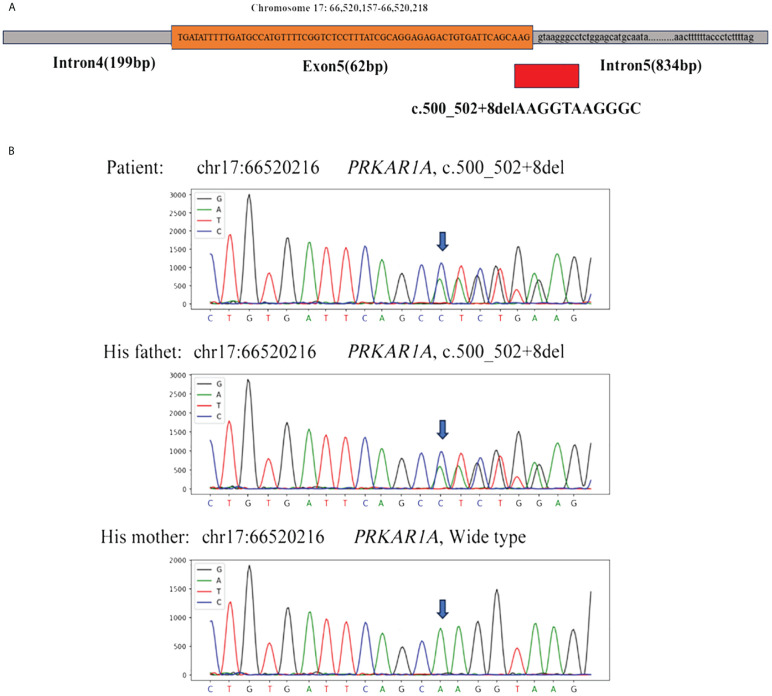
**(A)** Whole exome sequencing revealed a heterozygous deletion mutation in *PRKAR1A* (c.500_502 + 8del); **(B)** Sanger sequencing revealed that the patient and his father had the same deletion mutation at chr17:66520216 (c.500_502 + 8del), while the mother was wild type.

**Figure 4:**
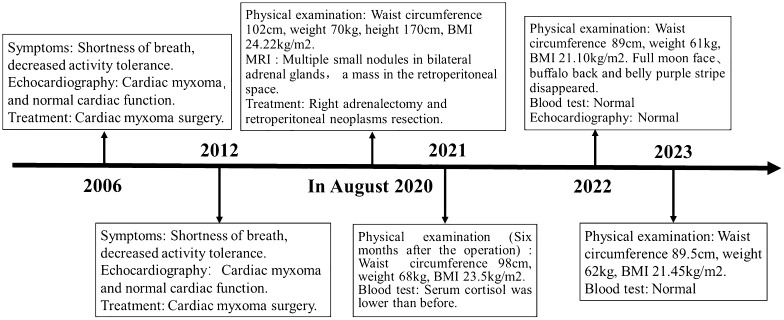
Case report of follow-up time and the corresponding symptoms, signs, examination and treatment results.

With the consent of the patient and his family, we collected peripheral blood samples from the patient and his parents for whole exome sequencing. The genome sequence report revealed that a novel c.500_502 + 8del in exon 5 of the *PRKAR1A* gene, situated on chromosome chr17:66520216 (**Figure 3**). According to the 2001 Stratakis CA Carney complex diagnostic criteria (12), the patient met the diagnostic criteria of labial nevus, cardiac myxoma, pigmented nodular adrenocortical disease (PPNAD), psammomatous melanotic schwannoma (PMS), and testicular calcification (**Table 2**), and was diagnosed with Carney complex. After long-term follow-up, the patient’s condition was stable, with weight loss, facial moon facies disappeared (**Figure 2E**), waist circumference reduction, significantly lower cortisol levels, and normal blood lipids (**Figure 4**, **Table 1**).

**Table 2:** Diagnostic criteria for CNC by Stratakis CA in 2001.

	Content
**Major criteria**	1. Spotty skin pigmentation in typical distribution.
	2. Cutaneous/mucosal or cardiac myxoma.
	3. Breast myxomatosis.
	4. PPNAD or paradoxically positive Liddle‘s test
	5. Acromegaly secondary to GH adenoma.
	6. Large calcified testicular tumor (LCCSCT) thyroid carcinoma.
	7. Psammomatous melanotic schwannoma (PMS).
	8. Multiple epithelioid blue nevus.
	9. Breast ductal adenoma.
	10. Osteochondromyxoma.
	11. Thyroid carcinoma or multiple hypoechoic nodules on thyroid ultrasonography
**Supplemental criteria**	1. Affected first – degree relative.
	2. Activating mutations of *PRKACA* and *PRKACB*.
	3. Inactivating mutation of *PRKAR1A* gene.
Diagnostic criteria	1. Consistent with two or more major criteria.
	2. Meets one or more major criteria, and at the same time, meets one or more of the supplementary criteria.

## 3 Discussion

In this article, we described a typical case of Carney complex, in which the patient’s clinical manifestations are characterized by non-ACTH-dependent Cushing’s syndrome, recurrent cardiac myxoma, psammomatous melanotic schwannoma (PMS) and skin mucosal lesions as the main clinical features. The patient’s father also has a history of cardiac myxoma, and genetic testing indicates a deletion mutation (c.500_502 + 8del) in the *PRKAR1A* gene for both the patient and the father. According to the Carney complex diagnostic criteria established by Stratakis CA in 2001 (12), the patient was diagnosed with Carney complex.

Carney complex is a rare autosomal dominant genetic syndrome caused mainly by mutations in the *PRKAR1A* gene, which encodes the regulatory subunit α of the cAMP-dependent protein kinase A (PKA) (3, 10). More than 130 different *PRKAR1A* variants have been found in more than 400 unrelated families, with most mutations occurring in a single family, among which nonsense mutations, frameshift mutations, and splicing site mutations account for the majority (1).Most *PRKAR1A* gene mutations result in nonsense-mediated mRNA decay (NMD), leading to loss or dysfunction of the RIα protein encoded by *PRKAR1A* and activation of the cAMP-PKA signaling pathway, which is considered the most common cause of the disease (13, 14). In this study, we report a novel *PRKAR1A* gene deletion mutation (c.500_502 + 8del) located in exon 5 at chromosome position chr17:66520216-66520226, and this deletion mutation has not been previously reported in cases of Carney complex. To gain further insights into the functional impact of this mutation, we utilized the MutationTaster database (https://mutationtaster.org/) (15). According to the database analysis, the c.500_502 + 8del mutation in *PRKAR1A* is predicted to be disease-causing. This prediction is based on the frameshift from the mutation, which alters splice sites and subsequently the amino acid sequence. These changes have the potential to influence the protein’s features and contribute to the development of the disease. The HSF (16) and SpliceAI (17) databases indicate that the c.500_502 + 8del mutation in the PRKAR1A gene disrupts the original Donor site, potentially affecting splicing. Thus, this mutation is related to the occurrence of Carney complex disease, and may alter the characteristics of proteins, contributing to the onset and progression of the patient’s condition. Carney complex has a high phenotypic variability, and the genotype-phenotype correlation is not yet clear. In this case, the same mutation was also found in the patient’s father, while his mother had the wild type. Interestingly, with the same mutation type, his father only exhibited cardiac myxomas and lip pigmentations, with the onset of cardiac myxomas occurring later in life. However, *PRKAR1A* gene mutation testing is helpful for the diagnosis of Carney complex. Therefore, Genetic testing is recommended for relatives of suspected or confirmed Carney complex patients.

The clinical manifestations of Carney complex are complex, mainly manifested as dysfunction of the endocrine system, various pigmented lesions of the skin and mucosa, and tumorous diseases. Endocrine over-activation is one of the main characteristics of the disease in patients with Carney complex. Patients with Carney complex may have dysfunction of various endocrine glands, including the pituitary, adrenal glands, thyroid glands, gonadal glands, and gastrointestinal tract. This leads to aberrant secretion of corresponding hormones ensues, giving rise to symptoms such as obesity, hyperlipidemia, osteoporosis (18) and hyperthyroidism (19). Primary pigmented nodular adrenocortical disease (PPNAD) represents the most prevalent endocrine tumor in Carney complex, with an incidence ranging from 25% to 60% (20). Pathologically, it is characterized by bilateral adrenal glands exhibiting multiple pigmented nodules accompanied by cortical atrophy. Clinical manifestations primarily encompass features associated with Cushing’s syndrome, including moon face, buffalo hump, acne, hirsutism, plethora, striae, and skin atrophy. Additionally, other endocrine and metabolic symptoms such as secondary amenorrhea, hypertension, and osteoporosis may also manifest. Long-term glucocorticoid replacement therapy after bilateral adrenalectomy is the classic method for PPNAD, but patients must receive lifelong glucocorticoid replacement therapy and adrenal crisis is prone to occur during treatment. Therefore, unilateral adrenalectomy has been reported in recent years as a safe and effective method for treating PPNAD, which can provide long-term relief of Cushing’s syndrome symptoms (21). However, some scholars believe that for patients with Carney complex who undergo unilateral adrenalectomy, retaining adrenal function may increase the risk of recurrence or incomplete cure. In this case, patient’s clinical symptoms improved significantly after unilateral adrenalectomy, but the blood cortisol remained elevated. Therefore, the surgical treatment plan for PPNAD patients should fully evaluate the condition of bilateral adrenal lesions, and for PPNAD patients who underwent unilateral adrenalectomy, close follow-up should be conducted after surgery to enhance their overall quality of life.

Myxoma is one of the common tumor types in Carney complex, which can occur in the heart (22), bone (23), skin (1)and other sites. Cardiac myxoma occurs in about 20-40% of Carney complex patients, and early Carney complex patients with cardiac myxoma as the first diagnosis are easily missed (24). Cardiac myxoma can occur in any one or more chambers of the heart, and its recurrence risk is as high as 22%, which is easy to lead to embolic stroke and heart failure, which significantly increases the mortality of Carney complex (25). In this case, the patient had cardiac myxoma as the first manifestation in the early stage, and the cardiac myxoma recurred after surgery. Brain tumor MRI showed bilateral paraventricular infarction, but fortunately did not lead to stroke. Therefore, prompt initiation of treatment is imperative for patients with cardiac myxoma, particularly those presenting at an early age, atypical location, multiple or recurrent case. Vigilance towards Carney complex-associated cardiac myxoma should be exercised. Once diagnosed, timely intervention measures must be implemented alongside regular follow-up using cardiac color Doppler ultrasound to significantly enhance patient prognosis and mitigate the risk of embolism and sudden death.

Skin lesions are the most prominent feature of patients with Carney complex, with more than 80% of patients having skin manifestations (26). The main clinical manifestations include freckles, pigmented nevus, and skin myxoma. Skin lesions can be surgically removed, and since they are benign tumors, follow-up observation is also possible. Psammomatous melanotic schwannoma (PMS) is a rare tumor in Carney complex. Pathologically, it is characterized by multicentricity, hyperpigmentation, and easy calcification, and is distributed in the digestive tract, paraspinal sympathetic nerve chain, and chest wall. It is characterized by pain and symptoms of nerve root lesions. Symptomatic patients should undergo MRI examinations of the brain, spine, chest, abdomen, pelvis, and peritoneum (27). In addition, Carney complex has also been reported in various tumor diseases, such as breast cancer (28), testicular tumor (29), and thyroid tumor (30, 31). Treatment plans are determined according to tumor location, size, function, benign and malignant, and clinical manifestations.

In summary, Carney complex is a rare multiple tumor syndrome characterized by pigmented lesions of the skin and mucosa, myxoma, and multiple endocrine and non-endocrine tumors. In this paper, we report a typical case of Carney complex in a patient who achieved a favorable prognosis through multidisciplinary systematic treatment. However, this study has some limitations. Firstly, after being diagnosed at our hospital, the patient chose to undergo surgical treatment elsewhere, resulting in insufficient information on the surgical procedures. Secondly, owing to the rarity of Carney complex, there is presently insufficient clinical data available for comparative analysis. Due to its rare and complex clinical manifestations, diagnosis is often delayed. Therefore, we advocate for comprehensive history taking, particularly past medical and family history, and thorough physical examinations for suspected Carney complex patients. Biochemical tests, imaging studies, histological examinations, and genetic testing should be conducted to aid in diagnosis. Once diagnosed, appropriate treatment measures should be taken for lesions affecting different systems, prioritizing those that are life-threatening and prognostically significant. For patients with Carney complex, we recommend long-term follow-up to improve quality of life and overall survival. Family members of patients diagnosed with Carney complex should undergo relevant screening, including endocrine evaluations, echocardiography, and genetic testing, to detect and treat potential lesions early. Furthermore, the development of Carney complex is closely related to mutations in the *PRKAR1A* gene. Developing targeted therapies against *PRKAR1A* mutations will aid in the treatment of Carney complex, and this will be the focus of our future research.

## Data availability statement

The raw data supporting the conclusions of this article will be made available by the authors, without undue reservation.

## Ethics statement

Written informed consent was obtained from the individual(s) for the publication of any potentially identifiable images or data included in this article.

## Author contributions

HZ: Data curation, Writing – original draft, Writing – review & editing. HK: Writing – review & editing. YQ: Formal Analysis, Writing – original draft. LX: Writing – review & editing. JW: Writing – review & editing. PL: Writing – review & editing. JK: Data curation, Supervision, Writing – original draft, Writing – review & editing.
